# Severe Pediatric Neurological Manifestations With SARS-CoV-2 or MIS-C Hospitalization and New Morbidity

**DOI:** 10.1001/jamanetworkopen.2024.14122

**Published:** 2024-06-10

**Authors:** Conall Francoeur, Alicia M. Alcamo, Courtney L. Robertson, Mark S. Wainwright, Juan D. Roa, Marlina E. Lovett, Casey Stulce, Mais Yacoub, Renee M. Potera, Elizabeth Zivick, Adrian Holloway, Ashish Nagpal, Kari Wellnitz, Katelyn M. Even, Werther Brunow de Carvalho, Isadora S. Rodriguez, Stephanie P. Schwartz, Tracie C. Walker, Santiago Campos-Miño, Leslie A. Dervan, Andrew S. Geneslaw, Taylor B. Sewell, Patrice Pryce, Wendy G. Silver, Jieru E. Lin, Wendy S. Vargas, Alexis Topjian, Jennifer L. McGuire, Jesus Angel Domínguez Rojas, Jaime Tasayco-Muñoz, Sue J. Hong, William J. Muller, Matthew Doerfler, Cydni N. Williams, Kurt Drury, Dhristie Bhagat, Aaron Nelson, Dana Price, Heda Dapul, Laura Santos, Robert Kahoud, Brian Appavu, Kristin P. Guilliams, Shannon C. Agner, Karen H. Walson, Lindsey Rasmussen, Ria Pal, Anna Janas, Peter Ferrazzano, Raquel Farias-Moeller, Kellie C. Snooks, Chung-Chou H. Chang, Tomás Iolster, Jennifer C. Erklauer, Facundo Jorro Baron, Evangeline Wassmer, Michael Yoong, Michelle Jardine, Zoha Mohammad, Akash Deep, Tanil Kendirli, Karen Lidsky, Samantha Dallefeld, Helen Flockton, Shruti Agrawal, Krishna Sumanth Siruguppa, Michaela Waak, Alfonso Gutiérrez-Mata, Warwick Butt, Sixto Bogantes-Ledezma, Fabricio Sevilla-Acosta, Andres Umaña-Calderón, Adriana Ulate-Campos, Adriana Yock-Corrales, Victor Brodzik Talisa, Hari Krishnan Kanthimathinathan, Michelle E. Schober, Ericka L. Fink

**Affiliations:** 1 Division of Pediatric Critical Care Medicine, Department of Pediatrics, Montreal Children’s Hospital, McGill University Health Center, Montreal, Quebec, Canada; 2 Division of Critical Care Medicine, Children’s Hospital of Philadelphia, Philadelphia, Pennsylvania; 3 Department of Anesthesiology and Critical Care, University of Pennsylvania Perelman School of Medicine, Philadelphia; 4 Department of Pediatrics, University of Pennsylvania Perelman School of Medicine, Philadelphia; 5 Department of Anesthesiology and Critical Care Medicine, Johns Hopkins University School of Medicine, Baltimore, Maryland; 6 Division of Pediatric Neurology, University of Washington, Seattle Children’s Hospital, Seattle; 7 Department of Pediatrics, Universidad Nacional de Colombia, Bogotá, Colombia; 8 Department of Pediatric Neurology, Faculty of Medicine, Fundación Universitaria de Ciencias de la Salud, Bogotá, Colombia; 9 Division of Critical Care Medicine, Department of Pediatrics, Nationwide Children’s Hospital, The Ohio State University, Columbus; 10 Department of Pediatrics, The University of Chicago, Chicago, Illinois; 11 Division of Critical Care, Department of Pediatrics, University Medical Center Children’s Hospital, Las Vegas, Nevada; 12 Department of Pediatrics, The University of Texas Southwestern Medical Center, Dallas; 13 Division of Pediatric Critical Care Medicine, Department of Pediatrics, Medical University of South Carolina, Charleston; 14 Division of Critical Care, Department of Pediatrics, University of Maryland Medical Center, Baltimore; 15 Department of Pediatrics, Section of Critical Care Medicine, Oklahoma Children’s Hospital at Oklahoma University Health, College of Medicine, The University of Oklahoma Health Sciences, Oklahoma City; 16 Division of Pediatric Critical Care, Department of Pediatrics, Carver College of Medicine, University of Iowa Health Care, Iowa City; 17 Division of Pediatric Critical Care Medicine, Penn State College of Medicine, The Pennsylvania State University, Hershey; 18 Department of Pediatrics, University of São Paulo, São Paulo, Brazil; 19 Department of Pediatrics, University of North Carolina at Chapel Hill Hospitals, Chapel Hill; 20 Pediatric Intensive Care Unit, Hospital Metropolitano, Quito, Ecuador; 21 Division of Pediatric Critical Care Medicine, Department of Pediatrics, University of Washington School of Medicine, Seattle; 22 Division of Pediatric Critical Care and Hospital Medicine, Department of Pediatrics, Columbia University Irving Medical Center, New York, New York; 23 Division of Pediatric Critical Care and Hospital Medicine, Department of Pediatrics, Columbia University Irving Medical Center, New York-Presbyterian Morgan Stanley Children’s Hospital, New York, New York; 24 Division of Child Neurology, Department of Neurology, Columbia University Irving Medical Center, New York, New York; 25 Division of Neurology, Children’s Hospital of Philadelphia, Philadelphia, Pennsylvania; 26 Department of Neurology, University of Pennsylvania Perelman School of Medicine, Philadelphia; 27 Division of Pediatric Critical Care, Department of Pediatrics, Hospital de Emergencia Villa El Salvador, Lima, Peru; 28 Department of Pediatrics, Ann & Robert H. Lurie Children’s Hospital of Chicago, Northwestern University Feinberg School of Medicine, Chicago, Illinois; 29 Pediatric Critical Care and Neurotrauma Recovery Program, Department of Pediatrics, Oregon Health & Science University, Portland; 30 Division of Pediatric Critical Care, Department of Pediatrics, Oregon Health & Science University, Portland; 31 Department of Neurology, New York University Langone Health, New York; 32 Division of Pediatric Critical Care, Department of Pediatrics, Hassenfeld Children’s Hospital, New York University Langone Health, New York; 33 Division of Pediatric Critical Care Medicine, Department of Pediatrics and Adolescent Medicine, Mayo Clinic College of Medicine and Science, Rochester, Minnesota; 34 Division of Neurology, Barrow Neurological Institute at Phoenix Children’s Hospital, The University of Arizona, College of Medicine, Phoenix; 35 Department of Neurology, Washington University School of Medicine in St Louis, St Louis, Missouri; 36 Edward Mallinckrodt Department of Pediatrics, Washington University School of Medicine in St Louis, St Louis, Missouri; 37 Mallinckrodt Institute of Radiology, Washington University School of Medicine in St Louis, St Louis, Missouri; 38 Division of Pediatric Critical Care Medicine, Emory University School of Medicine, Children’s Healthcare of Atlanta, Atlanta, Georgia; 39 Division of Pediatric Critical Care Medicine, Department of Pediatrics, Standford University Medicine, Lucile Packard Children’s Hospital Stanford, Stanford, California; 40 Safar Center for Resuscitation Research, University of Pittsburgh School of Medicine, UPMC Children’s Hospital of Pittsburgh, Pittsburgh, Pennsylvania; 41 Department of Pediatrics, University of Wisconsin School of Medicine and Public Health, Madison; 42 Division of Child Neurology, Department of Neurology, Medical College of Wisconsin, Children’s Wisconsin, Milwaukee; 43 Department of Pediatrics, Medical College of Wisconsin, Children’s Wisconsin, Milwaukee; 44 Division of Pediatric Critical Care Medicine, UPMC Children’s Hospital of Pittsburgh, Pittsburgh, Pennsylvania; 45 Department of Pediatrics, Hospital Universitario Austral, Buenos Aires, Argentina; 46 Division of Pediatric Critical Care Medicine, Department of Pediatrics, Baylor College of Medicine, Texas Children’s Hospital, Houston; 47 Division of Pediatric Neurology and Developmental Neuroscience, Department of Pediatrics, Baylor College of Medicine, Texas Children’s Hospital, Houston; 48 Department of Pediatrics, Hospital General de Niños Pedro Elizade, Buenos Aires, Argentina; 49 Birmingham Children’s Hospital, Birmingham, United Kingdom; 50 Aston Institute of Health and Neurodevelopment, Birmingham, United Kingdom; 51 Aston University, Birmingham, United Kingdom; 52 Department of Neurology, Royal London Children’s Hospital, London, United Kingdom; 53 Pediatric Critical Care Unit, University Hospital of Wales, Cardiff, United Kingdom; 54 Pediatric Intensive Care Unit, University Hospitals Leicester NHS Trust, Leicester, United Kingdom; 55 Department of Women and Children’s Health, King’s College Hospital, London, United Kingdom; 56 Department of Pediatric Critical Care Medicine, Ankara University School of Medicine, Ankara, Turkey; 57 Division of Pediatric Critical Care Medicine, Wolfson Children’s Hospital, Jacksonville, Florida; 58 Department of Pediatrics, Dell Children’s Hospital, Austin, Texas; 59 Paediatric Critical Care Unit, Oxford University Hospitals, Oxford, United Kingdom; 60 Paediatric Intensive Care Unit, Cambridge University Hospitals, Cambridge, United Kingdom; 61 Division of Pediatric Critical Care, Department of Pediatrics, University of California, San Francisco; 62 Fresno Medical Education and Research Program, Department of Medicine, University of California, San Francisco, Fresno; 63 Department of Pediatrics, Community Medical Centers, Fresno, California; 64 Child Health Research Centre, The University of Queensland, Brisbane, Queensland, Australia; 65 Department of Pediatric Neurology, Dr. Carlos Sáenz Herrera National Children’s Hospital, San José, Costa Rica; 66 Department of Critical Care, Melbourne Medical School, The University of Melbourne, The Royal Children’s Hospital Melbourne, Melbourne, Victoria, Australia; 67 Clinical Sciences, Murdoch Children’s Research Institute, Parkville, Victoria, Australia; 68 Department of Pediatrics, La Anexión Hospital, Guanacaste, Costa Rica; 69 Department of Emergency Service, Dr. Carlos Sáenz Herrera National Children’s Hospital, San José, Costa Rica; 70 West Midlands Regional Genetics Laboratory, Birmingham Women’s and Children’s NHS Foundation Trust, Birmingham, United Kingdom; 71 Division of Critical Care, Department of Pediatrics, University of Utah School of Medicine, Salt Lake City

## Abstract

**Importance:**

Neurological manifestations during acute SARS-CoV-2–related multisystem inflammatory syndrome in children (MIS-C) are common in hospitalized patients younger than 18 years and may increase risk of new neurocognitive or functional morbidity.

**Objective:**

To assess the association of severe neurological manifestations during a SARS-CoV-2–related hospital admission with new neurocognitive or functional morbidities at discharge.

**Design, Setting, and Participants:**

This prospective cohort study from 46 centers in 10 countries included patients younger than 18 years who were hospitalized for acute SARS-CoV-2 or MIS-C between January 2, 2020, and July 31, 2021.

**Exposure:**

Severe neurological manifestations, which included acute encephalopathy, seizures or status epilepticus, meningitis or encephalitis, sympathetic storming or dysautonomia, cardiac arrest, coma, delirium, and stroke.

**Main Outcomes and Measures:**

The primary outcome was new neurocognitive (based on the Pediatric Cerebral Performance Category scale) and/or functional (based on the Functional Status Scale) morbidity at hospital discharge. Multivariable logistic regression analyses were performed to examine the association of severe neurological manifestations with new morbidity in each SARS-CoV-2–related condition.

**Results:**

Overall, 3568 patients younger than 18 years (median age, 8 years [IQR, 1-14 years]; 54.3% male) were included in this study. Most (2980 [83.5%]) had acute SARS-CoV-2; the remainder (588 [16.5%]) had MIS-C. Among the patients with acute SARS-CoV-2, 536 (18.0%) had a severe neurological manifestation during hospitalization, as did 146 patients with MIS-C (24.8%). Among survivors with acute SARS-CoV-2, those with severe neurological manifestations were more likely to have new neurocognitive or functional morbidity at hospital discharge compared with those without severe neurological manifestations (27.7% [n = 142] vs 14.6% [n = 356]; *P* < .001). For survivors with MIS-C, 28.0% (n = 39) with severe neurological manifestations had new neurocognitive and/or functional morbidity at hospital discharge compared with 15.5% (n = 68) of those without severe neurological manifestations (*P* = .002). When adjusting for risk factors in those with severe neurological manifestations, both patients with acute SARS-CoV-2 (odds ratio, 1.85 [95% CI, 1.27-2.70]; *P* = .001) and those with MIS-C (odds ratio, 2.18 [95% CI, 1.22-3.89]; *P* = .009) had higher odds of having new neurocognitive and/or functional morbidity at hospital discharge.

**Conclusions and Relevance:**

The results of this study suggest that children and adolescents with acute SARS-CoV-2 or MIS-C and severe neurological manifestations may be at high risk for long-term impairment and may benefit from screening and early intervention to assist recovery.

## Key Points

**Question:**

Are severe pediatric neurological manifestations during a SARS-CoV-2–related hospitalization associated with new neurocognitive or functional morbidities?

**Findings:**

In this cohort study of 3568 patients younger than 18 years, hospitalized with acute SARS-CoV-2 or multisystem inflammatory syndrome in children, severe neurological manifestations, including acute encephalopathy, seizures or status epilepticus, and delirium, were common and were associated with new neurocognitive or functional morbidity at hospital discharge.

**Meaning:**

These findings suggest that patients under 18 years with a SARS-CoV-2–related hospitalization who experience severe neurological manifestations should be evaluated for new morbidity.

## Teaser

This cohort study of patients younger than 18 years hospitalized for SARS-CoV-2 or multisystem inflammatory syndrome evaluates the association of severe neurological manifestations with new neurocognitive or functional morbidities at discharge.

## Introduction

Most children infected with SARS-CoV-2 do not require hospitalization, and mortality rates are low.^1,2^ For some, however, acute SARS-CoV-2 infection can result in a significant burden of illness, especially among infants and children with comorbid illness.^3,4,5,6,7,8,9^ Children with the SARS-CoV-2–related condition multisystem inflammatory syndrome in children (MIS-C) often require critical care due to multisystem organ dysfunction, with high survival rates^10,11^ but with risk of post–critical illness sequelae. In particular, neurological manifestations of pediatric SARS-CoV-2–related conditions have been associated with morbidity and mortality in prior studies.^12,13^

Neurological manifestations of SARS-CoV-2–related conditions include a wide array of symptoms or conditions with varying severity.^12,13,14,15,16,17^ In our group’s multicenter preliminary report of neurological manifestations in hospitalized individuals younger than 18 years with SARS-CoV-2–related conditions, we found that 44% had at least 1 neurological manifestation (40% in patients with acute SARS-CoV-2 and 66% in patients with MIS-C).^12^ The most common symptoms were headache (16% in acute SARS-CoV-2 and 47% in MIS-C), which may be nonspecific and challenging to evaluate in younger children, and acute encephalopathy or altered mental status (15% in acute SARS-CoV-2 and 22% in MIS-C). To date, few studies have evaluated the potential association of neurological manifestations with outcomes, and only a limited number of symptoms and conditions have been considered.^17^

We performed a secondary analysis of an international, multicenter cohort study^12^ to describe severe neurological manifestations and analyze their association with new neurocognitive or functional morbidity at hospital discharge. We hypothesized that the occurrence of severe neurological manifestations during acute SARS-CoV-2 or MIS-C hospitalization would be associated with new neurocognitive and/or functional morbidity at hospital discharge compared with individuals under 18 years without severe neurological manifestation. An association between severe neurological manifestations and new neurocognitive and/or functional morbidity may suggest severe neurological manifestations as an independent predictor of poor outcomes in hospitalized young patients with acute SARS-CoV-2 or MIS-C and therefore could be used to guide postdischarge management.

## Methods

### Study Population

This cohort study is a secondary analysis of individuals younger than 18 years with acute SARS-CoV-2 or SARS-CoV02–related MIS-C with and without severe neurological manifestations from the pediatric Global Consortium Study of Neurologic Dysfunction in COVID-19 (GCS-NeuroCOVID).^12^ The GCS-NeuroCOVID was a prospective, multicenter, international cohort study performed in 46 centers across 10 countries. Patients under the age of 18 years who were admitted with acute SARS-CoV-2–related MIS-C were included, and only those previously enrolled were excluded. As described in the initial GCS-NeuroCOVID,^12^ acute SARS-CoV-2 was classified as either confirmed (a positive SARS-CoV-2 test result) or presumed (a clinical diagnosis in the setting of clinical suspicion and/or close contact) without a diagnosis of MIS-C. An MIS-C diagnosis was classified by the Centers for Disease Control and Prevention definition.^12^ Patients were enrolled between January 2, 2020, and July 31, 2021. The enrollment period was extended beyond the preliminary report for the original sites from May 1, 2021, to July 31, 2021, and additional sites were added for the entire reporting period.^12^ Local regulatory approval was obtained at each study site, and the need for informed consent was determined by each site’s regulatory authority. The University of Pittsburgh Institutional Review Board was the main study site, and it approved the study. The data coordinating center that received and analyzed data was at the University of Pittsburgh. This study followed the Strengthening the Reporting of Observational Studies in Epidemiology (STROBE) reporting guideline.

### Data Collection

Data were collected from each study site using a case report form with common data elements and a data dictionary as previously described.^12^ Demographic, illness, laboratory, imaging, and outcomes data were collected. Race and ethnicity data, as defined by each site and reported in the medical records, were collected because of reported associations with COVID-19 outcomes.^18^ Race categories included Black, White, and other (including American Indian or Alaska Native, Asian, Native Hawaiian or Other Pacific Islander, and all other races), and ethnicity categories included Hispanic or Latino and non-Hispanic or non-Latino.

Neurological manifestations were previously defined and included a broad spectrum of symptoms from headache and malaise to encephalopathy and coma.^12,19^ For this study, severe neurological manifestations were defined during the design phase and before the primary analysis and were approved by expert opinion including consensus (by C.F., A.M.A., C.L.R., M.S.W., J.D.R., M.E.S., and E.L.F.) and evidence of association with worse outcomes in both SARS-CoV-2–related conditions and other acute pediatric illnesses.^13,20^ The final definition of severe manifestations included acute encephalopathy (altered mental status, lethargy, or drowsiness), seizures or status epilepticus, meningitis or encephalitis, sympathetic storming or dysautonomia, hypoxic ischemic brain injury secondary to cardiac arrest, coma, delirium, and stroke. These terms were indicated on the primary case report form. Laboratory values, including liver enzymes, inflammatory markers (eg, interleukin 6, C-reactive protein, ferritin, and procalcitonin), complete blood count, and electrolytes, were collected.

### Outcomes

The primary outcome was new neurocognitive and/or functional morbidity at hospital discharge. Neurocognitive morbidity was as defined as a Pediatric Cerebral Performance Category (PCPC) score of 3 or more with a change of 1 or more points from the prehospitalization baseline.^21^ Scores for the PCPC range from 1 to 6 (1 indicates normal; 2, mild disability; 3, moderate disability; 4, severe disability; and 5, for vegetative state or coma—6 indicates death, which was not included in the baseline scores but was possible after hospitalization and was used to define unfavorable outcomes). Functional morbidity was defined as a change in the Functional Status Scale score of 1 or more between baseline and hospital discharge scores.^22^ The Functional Status Scale is based on 6 domains of functioning, and each domain ranges from 1 to 5. The final score is a cumulative score of all 6 domains, with higher scores indicating more severe dysfunction. Secondary outcomes included hospital mortality and new neurocognitive morbidity alone or new functional morbidity alone (rather than a combined outcome).

### Statistical Analysis

Data were analyzed using Stata, version 18 (StataCorp LLC). Analyses were performed overall and by acute SARS-CoV-2 or MIS-C group, given the distinct differences in pathophysiology between these disease presentations.^11^ Categorical data were summarized as frequencies using χ^2^ tests and continuous data as median with IQR using Mann-Whitney tests. Multivariable logistic regression modeling was performed to evaluate the independent association of severe neurological manifestations during hospital admission with the presence of new neurocognitive and/or functional morbidity at hospital discharge for survivors. This regression analysis was stratified on and conducted within SARS-CoV-2 and MIS-C cohorts, and potential risk factors were considered separately in these populations.

We included age and sex a priori as potential risk factors for model building. The remaining demographic and clinical variables associated with new neurocognitive or functional morbidity at hospital discharge on univariable logistic regression with a *P* value <.20 were included as potential risk factors in model building. The final model was determined using backward stepwise elimination with a *P* value cutoff of .20 for removal from the model. A 2-sided *P* < .05 was considered statistically significant. Missing data were not imputed.

## Results

### Overall Patient Characteristics

During the study period, 3568 patients younger than 18 years were admitted to participating hospitals for either acute SARS-CoV-2 or MIS-C. The median age was 8 years (IQR, 1-14 years), 1627 (45.6%) were female, 3 (0.1%) were intersex, 1938 (54.3%) were male (n = 1938), and 1517 (42.5%) had no preexisting comorbidities. Of the total patients, 820 (23.0%) were Black, 1024 (28.7%) were Hispanic or Latino, 2271 (63.6%) were non-Hispanic or non-Latino, 1925 (54.0%) were White, and 890 (24.9%) were categorized as other race.

### Patient Characteristics by Acute SARS-CoV-2 and MIS-C Group

Most patients included in this study had acute SARS-CoV-2 (83.5% [n = 2980]), and the remainder had MIS-C (588 [16.5%]). Among the patients with acute SARS-CoV-2, 536 (18.0%) had a severe neurological manifestation during hospitalization, as did 146 (24.8%) of the patients with MIS-C. In patients with acute SARS-CoV-2, those with severe neurological manifestations were less likely to be Hispanic or Latino (26.7% vs 30.4%), more likely to have more than 1 preexisting comorbidity (46.6% vs 32.5%), and more likely to have a neurological preexisting condition (45.7% vs 16.3%) compared with those without severe neurological manifestations (eTable 1 in Supplement 1). In patients with MIS-C, those with severe neurological manifestations were more likely to have a preexisting neurological condition (11.6% vs 5.9%) compared with those without severe neurological manifestations (Table 1).

**Table 1.** Clinical Characteristics of Patients With and Without Severe Neurological Manifestations by Acute SARS-CoV-2 and MIS-C Group^a^

Characteristic	Patients with SARS-CoV-2, No. (%)		Patients with MIS-C, No. (%)	
	Without severe neurological manifestations (n = 2444)	With severe neurological manifestations (n = 536)	Without severe neurological manifestations (n = 442)	With severe neurological manifestations (n = 146)
Age, median (IQR)^b^	8.0 (0.9-14.0)	7.0 (1.1-14.0)	8.5 (2.0-12.0)	8.6 (5.0-12.0)
Sex				
Female	1145 (46.8)	227 (42.4)	193 (43.7)	62 (42.5)
Intersex	3 (0.1)	0	0	0
Male	1296 (53.0)	309 (57.6)	249 (56.3)	84 (57.5)
Race				
Black	553 (22.6)	101 (18.8)	123 (27.8)	43 (29.5)
White	1283 (52.5)	301 (56.2)	208 (47.1)	66 (45.2)
Other^c^	608 (24.9)	134 (25.0)	111 (25.1)	37 (25.3)
Ethnicity				
Hispanic or Latino	743 (30.4)	143 (26.7)	107 (24.2)	31 (21.2)
Non-Hispanic or non-Latino	1504 (61.5)	363 (67.7)	296 (67.0)	108 (74.0)
Not reported	197 (8.1)	30 (5.6)	39 (8.8)	7 (4.8)
Type of comorbidity				
None	955 (39.1)	156 (29.1)	308 (69.7)	98 (67.1)
Neurological	398 (16.3)	245 (45.7)	26 (5.9)	17 (11.6)
Nonneurological	1091 (44.6)	135 (25.2)	108 (24.4)	31 (21.2)
Total comorbidities				
0	955 (39.1)	156 (29.1)	308 (69.7)	98 (67.1)
1	695 (28.4)	130 (24.3)	95 (21.5)	24 (16.4)
>1	794 (32.5)	250 (46.6)	39 (8.8)	24 (16.4)
Baseline PCPC				
1	1989 (81.4)	317 (59.1)	404 (91.4)	126 (86.3)
2	137 (5.6)	54 (10.1)	12 (2.7)	6 (4.1)
3	84 (3.4)	52 (9.7)	3 (0.7)	7 (4.8)
4	91 (3.7)	72 (13.4)	2 (0.5)	5 (3.4)
5	0	0	0	1 (0.7)
Missing	143 (5.9)	41 (7.6)	21 (4.8)	1 (0.7)
Baseline FSS				
6-7	1950 (79.8)	344 (64.2)	388 (87.8)	129 (88.4)
8-9	90 (3.7)	27 (5.0)	4 (0.9)	4 (2.7)
10-14	73 (3.0)	53 (9.9)	0	2 (2.7)
≥15	37 (1.5)	25 (4.7)	1 (0.2)	1 (0.7)
Missing	294 (12.0)	87 (16.2)	49 (11.11)	8 (5.5)
Pandemic epoch				
January-June 2020	431 (17.6)	90 (16.8)	55 (12.4)	24 (16.4)
July-December 2020	1131 (46.3)	256 (47.8)	176 (39.8)	66 (45.2)
January-July 2021	725 (29.7)	164 (30.6)	201 (43.5)	52 (35.6)
Missing	157 (6.4)	26 (4.9)	10 (2.3)	4 (2.7)
ICU admission	1316 (53.8)	310 (57.8)	213 (48.2)	68 (46.6)
Neurological manifestation				
Acute encephalopathy	NA	332 (61.9)	NA	111 (76.0)
Seizures or status epilepticus	NA	218 (40.7)	NA	14 (9.6)
Delirium	NA	40 (7.5)	NA	17 (11.6)
Coma	NA	40 (7.5)	NA	7 (4.8)
Hypoxic ischemic brain injury secondary to cardiac arrest	NA	36 (6.7)	NA	8 (5.5)
Meningitis or encephalitis	NA	28 (5.2)	NA	12 (8.2)
Stroke	NA	14 (2.6)	NA	5 (3.4)
Sympathetic storming or dysautonomia	NA	32 (5.9)	NA	16 (10.9)

Abbreviations: FSS, Functional Status Scale; ICU, intensive care unit; MIS-C, multisystem inflammatory syndrome in children; NA, not applicable; PCPC, Pediatric Cerebral Performance Category.

^a^ Scoring for the PCPC and FSS are described in Methods.

^b^ Age was not recorded for all patients: number without acute SARS-CoV-2, 2438; with acute SARS-CoV-2, 535; without MIS-C, 442; and with MIS-C, 145.

^c^ Other includes American Indian or Alaska Native, Asian, Native Hawaiian or Other Pacific Islander, and all other races.

The most common severe neurological manifestations with acute SARS-CoV-2 were acute encephalopathy (332 patients [61.9%]), seizures or status epilepticus (218 patients [40.7%]), and delirium and coma (both 40 patients [7.5%]). For MIS-C, the most common severe neurological manifestations were acute encephalopathy (111 patients [76.0%]), delirium (17 patients [11.6%]), sympathetic storming or dysautonomia (16 patients [10.9%]), and seizures or status epilepticus (14 patients [9.6%]) (Table 1).

### Outcomes by Acute SARS-CoV-2 and MIS-C Group

#### SARS-CoV-2 Group

Of patients with severe neurological manifestations in acute SARS-CoV-2, 24 (4.8%) died within the hospitalization compared with 7 (0.3%) of those without severe neurological manifestations. Survivors with acute SARS-CoV-2 who experienced severe neurological manifestations were more likely to have new neurocognitive and/or functional morbidity at hospital discharge compared with those without severe neurological manifestations (27.7% [n = 142] vs 14.6% [n = 356]; *P* < .001). New neurocognitive morbidity was identified in 17.9% (n = 92) vs 7.1% (n = 173) of survivors with acute SARS-CoV-2 who experienced severe neurological manifestations compared with those who did not experience severe neurological manifestations, and new functional morbidity was identified in 26.7% (n = 137) vs 14.2% (n = 347) of those with and without severe neurological manifestations, respectively (both *P* < .001). Furthermore, patients who had severe neurological manifestations during acute SARS-CoV-2 were more likely to receive physical therapy, occupational therapy, speech and language therapy, and rehabilitation consults during hospitalization compared with those without severe neurological manifestations (Table 2).

**Table 2.** Hospital Outcomes for Patients With and Without Severe Neurological Manifestations by Acute SARS-CoV-2 and MIS-C Group

Outcome	Patients with SARS-CoV-2, No. (%)		*P* value	Patients with MIS-C, No. (%)		*P* value
	Without severe neurological manifestations (n = 2444)	With severe neurological manifestations (n = 536)		Without severe neurological manifestations 442)	With severe neurological manifestations (n = 146)	
Hospital mortality	7 (0.3)	24 (4.8)	<.001	2 (0.5)	7 (4.9)	<.001
New neurocognitive and/or functional morbidity^a^	356 (14.6)	142 (27.7)	<.001	68 (15.5)	39 (28.0)	.002
New neurocognitive morbidity^a^	173 (7.1)	92 (17.9)	<.001	33 (7.5)	23 (16.5)	.003
New functional morbidity^a^	347 (14.2)	137 (26.7)	<.001	63 (14.3)	32 (23.0)	.03
Consults						
Physical therapy	243 (11)	130 (27.8)	<.001	122 (30.4)	63 (46.3)	<.001
Occupational therapy	164 (7.4)	103 (22.0)	<.001	87 (21.8)	47 (34.8)	.003
Speech and language therapy	72 (3.3)	57 (12.2)	<.001	15 (3.8)	24 (17.8)	<.001
Rehabilitation	31 (1.7)	39 (8.3)	<.001	9 (2.2)	12 (8.5)	<.001
Hospital length of stay, median (IQR), d^b^	3.0 (2.0-6.0)	4.0 (2.0-11.0)	<.001	6.0 (5.0-9.0)	8.0 (5.0-14.0)	<.001

Abbreviation: MIS-C, multisystem inflammatory syndrome in children.

^a^ Frequency calculated in survivors only.

^b^ Because hospital length of stay was not recorded for all patients, the number of patients without acute SARS-CoV-2 was 2440; with acute SARS-CoV-2, 533; without MIS-C, 441; and with MIS-C, 144.

#### MIS-C Group

Among patients with MIS-C, those with severe neurological manifestations had higher in-hospital mortality (4.9% [n = 7]) compared with those without severe neurological manifestations (0.5% [n = 2]) (*P* < .001). Among survivors with MIS-C, 28.0% (n = 39) of those with severe neurological manifestations had new neurocognitive and/or functional morbidity at hospital discharge compared with 15.5% (n = 68) of those without severe neurological manifestations (*P* = .002). New neurocognitive morbidity was identified in 16.5% (n = 23) vs 7.5% (n = 33) (*P* = .003) and new functional morbidity in 23.0% (n = 32) vs 14.3% (n = 63) (*P* = .03) of those with severe neurological manifestations compared with those without severe neurological manifestations. Patients with MIS-C who had severe neurological manifestations were more likely to receive physical therapy, occupational therapy, speech and language therapy, and rehabilitation consults during hospitalization compared with those without (Table 2).

### Laboratory Values and Neurodiagnostics by Acute SARS-CoV-2 and MIS-C Group

Patients with severe neurological manifestations had lower median (IQR) platelet counts on admission than did those without severe neurological manifestations for both the acute SARS-CoV-2 group (215.0 × 10^3^/μL [IQR, 133.0-302.5 × 10^3^/μL] vs 250.0 × 10^3^/μL [IQR, 171.0-331.0 × 10^3^/μL]; *P* < .001) and MIS-C group (114.0 × 10^3^/μL [IQR, 73.0-174.0 × 10^3^/μL] vs 143.0 × 10^3^/μL [IQR, 103.0-205.0 × 10^3^/μL]; *P* < .001) (to convert ×10^3^/μL to ×10^9^/L, multiply by 1.0). There was no difference in the C-reactive protein levels, lymphocyte counts, or sodium levels (eTable 2 in Supplement 1). For the patients with acute SARS-CoV-2, those with severe neurological manifestations more frequently received electroencephalography (26.1% vs 1.1%; *P* < .001), brain computed tomography (30.2% vs 3.7%; *P* < .001), and brain magnetic resonance imaging (20.6% vs 2.4%; *P* < .001) than did those without severe neurological manifestations. For the patients with MIS-C, those with severe neurological manifestations more frequently received electroencephalography (17.1% vs 1.4%; *P* < .001), brain computed tomography (23.6% vs 5.4%; *P* < .001), and brain magnetic resonance imaging (17.9% vs 2.9%; *P* < .001) than those without severe neurological manifestations (eTable 2 in Supplement 1).

### Treatments by Acute SARS-CoV-2 and MIS-C Group

Individuals with acute SARS-CoV-2 and severe neurological manifestations were more likely to receive remdesivir (14.9% vs 8.6%; *P* < .001), corticosteroids (22.0% vs 12.5; *P* < .001), and intravenous immunoglobulin (IVIG) treatments (2.8% vs 1.0%; *P* = .001) than those without severe neurological manifestations (eTable 2 in Supplement 1). In patients with MIS-C, those with severe neurological manifestations were more likely to receive remdesivir (6.2% vs 2.5%; *P* = .03) than were those without severe neurological manifestations, but both groups were similarly likely to receive corticosteroids or IVIG.

### Association Between Severe Neurological Manifestations and New Neurocognitive and/or Functional Morbidity at Discharge

For patients admitted with acute SARS-CoV-2 who survived the admission, those with severe neurological manifestations had higher odds of having new neurocognitive and/or functional morbidity at hospital discharge (odds ratio, 1.85 [95% CI, 1.27-2.70]; *P* = .001), adjusting for race, neurological comorbidity, corticosteroid administration, pandemic epoch, lymphocyte counts, and initial sodium levels (Table 3). Similarly, for surviving patients with MIS-C, those with severe neurological manifestations had higher odds of having new neurocognitive and/or functional morbidity at hospital discharge (odds ratio, 2.18 [95% CI, 1.22-3.89]; *P* = .009), adjusting for race, baseline PCPC score, IVIG administration, and initial sodium levels (Table 4).

**Table 3.** Multivariable Logistic Regression Model for New Neurocognitive and/or Functional Morbidity Based on the Presence of Severe Neurological Manifestations With Acute SARS-CoV-2

Characteristic	Odds ratio (95% CI)	*P* value
Severe neurological manifestation	1.85 (1.27-2.70)	.001
Black race	2.90 (2.07-4.07)	<.001
Neurological comorbidity	1.87 (1.29-2.70)	.001
Corticosteroid treatment	1.63 (1.09-2.42)	.02
Pandemic epoch July-December 2020	1.26 (1.03-1.12)	.18
Absolute lymphocyte count	1.00 (1.00-1.00)	.02
Initial sodium level	1.07 (1.02-1.12)	.002

**Table 4.** Multivariable Logistic Regression Model for New Neurocognitive or Functional Morbidity Based on the Presence of Severe Neurological Manifestations With MIS-C

Characteristic	Odds ratio (95% CI)	*P* value
Severe neurological manifestation	2.18 (1.22-3.89)	.009
Black race	2.52 (1.42-4.47)	.002
Baseline PCPC	5.47 (0.98-30.57)	.05
Intravenous immunoglobulin	0.60 (0.28-1.27)	.18
Initial sodium level	1.04 (0.98-1.1)	.18

Abbreviations: MIS-C, multisystem inflammatory syndrome in children; PCPC, Pediatric Cerebral Performance Category.

## Discussion

This international, multicenter cohort study of hospitalized individuals younger than 18 years with SARS-CoV-2–related conditions found that severe neurological manifestations were common in those admitted with acute SARS-CoV-2 (18.0%) or MIS-C (24.8%). Acute encephalopathy, seizure or status epilepticus, and delirium were among the most common severe manifestations in patients with both acute SARS-CoV-2 and MIS-C. For those with acute SARS-CoV-2 or MIS-C, the occurrence of a severe manifestation was associated with increased odds of new neurocognitive and/or functional morbidity at hospital discharge. The individuals with severe manifestations were also more likely to receive inpatient services including physical and occupational therapy and rehabilitation consults. These patients may be at risk for ongoing postdischarge sequelae.

Central nervous system involvement in acute SARS-CoV-2 or MIS-C carries potential for both short-term and long-term morbidities. Young persons who are hospitalized and critically ill can have significant morbidity after hospital discharge,^23,24,25^ including physical^26^ and neurocognitive and/or psychosocial dysfunctions.^25^ In our study, patients with severe neurological manifestations were more likely to have new neurocognitive and functional morbidities at hospital discharge. These patients were also more likely to receive physical therapy, occupational therapy, speech and language therapy, and rehabilitation consults during hospitalization. The finding that patients with severe neurologic manifesations were more likely to have new neurocognitive and functional morbidities at hospital discharge parallels observations in other pediatric critical illnesses. Among children with sepsis, the presence of neurological dysfunction is associated with greater odds of death or new moderate disability at discharge in survivors.^27^ Identifying the patients at the highest risk of new and persistent impairment is essential to direct them to the follow-up care necessary to best support their recovery and adaptation. Low platelet count, for example, found in both of the groups with severe neurological manifestations, may be a marker for disease severity in both acute SARS-CoV-2^28,29^ and MIS-C^30,31^ and therefore may serve as a flag for patients deserving scrutiny acutely and in follow-up. Despite strong agreement about the importance of postdischarge follow-up care of children who are critically ill, few systematic, multidisciplinary post–pediatric intensive care unit (ICU) follow-up programs exist,^32^ often due to logistical and reimbursement challenges. By identifying cohorts of patients at high risk of postdischarge sequelae, future research could demonstrate the value of these programs and their potential effects on improving long-term outcomes.

For both acute SARS-CoV-2 and MIS-C, patients with severe neurological manifestations were more likely to have a preexisting neurological condition. These findings are in agreement with a national study in the United Kingdom which found that children with medical comorbidities and/or neurodisability were at higher risk of pediatric ICU admission with severe COVID-19.^33^ Similarly, there is an association between short-term and long-term outcomes in patients with preexisting neurological conditions following ICU discharge for other illnesses. For example, children with neurodisability are at increased risk of ICU delirium,^34,35,36^ and children with ICU delirium are at increased risk of postdischarge decline in health-related quality of life.^37,38^ Preexisting neurological conditions are also a risk factor for the development of severe neurological manifestations of other virus-induced acute pediatric disease processes. For example, several studies found that children with influenza-associated neurological manifestations were more likely to have preexisting neurological comorbidity.^20,39,40,41^ Interestingly, in our MIS-C cohort, it was more common for patients without known comorbidities to have severe neurological manifestations. Some data do show, however, that despite initial severe illness, patients younger than 18 years with MIS-C have minimal functional impairment at 6-month follow-up.^42^ Our findings highlight the risk of new neurological manifestations among individuals in this age group with preexisting neurological conditions who present with either acute SARS-CoV-2 or MIS-C. Future studies of patients with acute SARS-CoV-2 and MIS-C, and likely those with other acute viral illnesses as well, should include and comprehensively evaluate those with preexisting neurological conditions for both in-hospital and postdischarge outcomes.

### Strengths and Limitations

Strengths of our study include its large multicenter, multinational prospective design and comprehensive data collection. In contrast to several large studies evaluating neurological complications of acute SARS-CoV-2 or MIS-C in children,^11,13,17,33,43,44,45^ our data included granular neurological outcomes at hospital discharge.

Our study also has limitations. Neurological manifestations were characterized based on a review of the medical record, and it is possible that these findings underestimated or overestimated their true prevalence. Some neurological diagnoses such as encephalopathy are more difficult to assess in individuals of different ages and developmental stages. We were unable to accurately ascertain the number of patients who incidentally tested positive for SARS-CoV-2 during hospitalization for other primary diagnoses. We also could not determine how many patients were not captured due to institutional differences in admission testing for SARS-CoV-2. In patients with preexisting neurological disease, we could not determine whether new neurological manifestations were secondary to SARS-CoV-2 disease presentation or to the preexisting neurological disease. There were imbalances in several covariates at baseline between groups. We collected data at hospital discharge, thus our study could not evaluate the long-term implications of these neurological manifestations, including the potential role of long COVID. Future studies should aim to address these limitations.

## Conclusions

This cohort study found that severe neurological manifestations were common in children hospitalized with acute SARS-CoV-2 and MIS-C and were associated with new neurocognitive and/or functional morbidities at hospital discharge compared with those without severe neurological manifestations. Patients with severe neurological manifestations with acute SARS-CoV-2 or MIS-C were more likely to have a preexisting neurological condition. Those with either acute SARS-CoV-2 or MIS-C who had severe neurological manifestations were more likely to require additional inpatient resources like physical and occupational therapy and rehabilitation consults. Future studies should aim to better understand the pathophysiology behind the severe neurological manifestations and to investigate the role of surveillance, treatment, and follow-up of these patients with high risk of neurocognitive and/or functional morbidities.

## Notes

Supplement 1. **eTable 1.** Comorbidities by Acute SARS-CoV-2 and MIS-C Groups **eTable 2.** Laboratory, Neurotesting, and Treatment Variables of Patients With and Without Severe Neurologic Manifestations by Acute SARS-CoV-2 and MIS-C Groups

Supplement 2.
Data Sharing Statement
