# Byline Errors

**DOI:** 10.1001/jamanetworkopen.2024.28458

**Published:** 2024-07-19

The Original Investigation “Severe Pediatric Neurological Manifestations With SARS-CoV-2 or MIS-C Hospitalization and New Morbidity,”^1^ published June 10, 2024, has been corrected to update the academic degrees of coauthors Wendy G. Silver, MD, MA, and Wendy S. Vargas, MD.
