# Supplementary material for: Severe Pediatric Neurological Manifestations With SARS-CoV-2 or MIS-C Hospitalization and New Morbidity
Source: JAMA Netw Open. 2024 Jun 10;7(6):e2414122. doi: 10.1001/jamanetworkopen.2024.14122 (PMC11165382; doi:10.1001/jamanetworkopen.2024.14122)
Supplement: Supplement 1. — eTable 1. Comorbidities by Acute SARS-CoV-2 and MIS-C Groups eTable 2. Laboratory, Neurotesting, and Treatment Variables of Patients With and Without Severe Neurologic Manifestations by Acute SARS-CoV-2 and MIS-C Groups [file jamanetwopen-e2414122-s001.pdf]

Supplemental Online Content

Francoeur C, Alcamo AM, Robertson CL, et al. Severe pediatric neurological manifestations with SARS-CoV-2 or MIS-C hospitalization and new morbidity. *JAMA Netw Open*. 2024;7(6):e2414122. doi:10.1001/jamanetworkopen.2024.14122

eTable 1. Comorbidities by Acute SARS-CoV-2 and MIS-C Groups
eTable 2. Laboratory, Neurotesting, and Treatment Variables of Patients With and Without Severe Neurologic Manifestations by Acute SARS-CoV-2 and MIS-C Groups

This supplemental material has been provided by the authors to give readers additional information about their work.

© 2024 Francoeur C et al. *JAMA Network Open*.

eTable 1. Comorbidities by Acute SARS-CoV-2 and MIS-C Groups

	**Acute SARS-CoV-2** Severe Neurologic Manifestation (No (%))			**MIS-C** Severe Neurologic Manifestation (No (%))		
**Comorbidity**	Absent (n=2444)	Present (n=536)	*P* value	Absent (n=442)	Present (n=146)	*P* value
Neurologic	398 (16.4)	245 (46.1)	<0.001	26 (5.9)	17 (11.6)	0.02
Cardiovascular	227 (9.3)	56 (10.5)	0.38	16 (3.6)	8 (5.5)	0.32
Respiratory	490 (20.1)	129 (24.3)	0.03	41 (9.3)	22 (15.2)	0.05
Renal or Urologic	148 (6.1)	29 (5.5)	0.59	14 (3.2)	7 (4.8)	0.35
Gastrointestinal	359 (14.7)	107 (20.2)	0.002	15 (3.4)	5 (3.5)	0.96
Hematologic or Immunologic	281 (11.5)	35 (6.6)	<0.001	16 (3.6)	8 (5.5)	0.32
Metabolic	221 (9.1)	69 (13.0)	0.006	19 (4.3)	7 (4.8)	0.79
Congenital or Genetic	323 (13.3)	105 (19.8)	<0.001	13 (2.9)	6 (5.1)	0.48
Oncological	138 (5.7)	30 (5.7)	0.97	6 (1.5)	1 (0.7)	0.47
Prematurity	219 (9.6)	78 (15.9)	<0.001	10 (2.4)	8 (5.8)	0.06
Technological Dependence	126 (5.2)	67 (12.6)	<0.001	4 (0.9)	4 (2.8)	0.09
Transplantation	59 (2.4)	10 (1.9)	0.44	2 (0.5)	1 (0.7)	0.78
Other	241 (10.0)	72 (13.6)	0.01	29 (6.6)	5 (3.4)	0.43

© 2024 Francoeur C et al. *JAMA Network Open*.

eTable 2. Laboratory, Neurotesting, and Treatment Variables of Patients With and Without Severe Neurologic Manifestations by Acute SARS-CoV-2 and MIS-C Groups

	**Acute SARS-CoV-2** Severe Neurologic Manifestation (No (%))			**MIS-C** Severe Neurologic Manifestation (No (%))		
**Variable**	Absent (n=2,444)	Present (n=536)	*P* value	Absent (n=442)	Present (n=146)	*P* value
**Laboratory**						
Highest C-reactive protein, mg/dL (median, [IQR])	3.0 [0.6, 13.4] (n=1,304)	5.0 [0.6, 18.5] (n=336)	0.079	20.9 [14.1, 25.6] (n=432)	23.6 [12.1, 40.9] (n=139)	0.47
Lowest Absolute Lymphocytes (median, [IQR])	1,318.0 [532.0, 2730.0] (n=1,878)	1,310.0 [438.5, 2640.0] (n=462)	0.62	580.0 [265.5, 995.0] (n=436)	483.0 [235.0, 1100.0] (n=144)	0.36
Lowest Platelets, x 10^3 (median, [IQR])	250.0 [171.0, 331.0] (n=1,966)	215.0 [133.0, 302.5] (n=484)	<0.001	143.0 [103.0, 205.0] (n=439)	114.0 [73.0, 174.0] (n=144)	<0.001
Initial Sodium, mMol/L (median, [IQR])	138.0 [136.0, 140.0] (n=1,838)	138.0 [136.0, 141.0] (n=482)	0.10	134.0 [132.0, 137.0] (n=353)	134.0 [131.0, 136.0] (n=133)	0.28
Highest Ferritin ng/mL (median, [IQR])	191.0 [85.0, 506.0] (n=593)	267.2 (79.0, 816.0] (n=185)	0.09	612.0 [338.7, 1118.0] (n=425)	869 [426.0, 1459.0) (n=139)	0.004
**Neurotesting**						
EEG	26 (1.1)	136 (26.1)	<0.001	6 (1.4)	24 (17.1)	<0.001
Abnormal EEG[a]	14 (53.8)	103 (75.7)	0.015	5 (83.3)	21 (87.5)	0.79
Brain CT	86 (3.7)	153 (30.2)	<0.001	24 (5.4)	34 (23.6)	<0.001
Abnormal CT [b]	39 (45.3)	69 (45.1)	0.87	4 (16.7)	9 (26.5)	0.34
Brain MRI	56 (2.4)	103 (20.6)	<0.001	13 (2.9)	26 (17.9)	<0.001
Abnormal MRI [c]	28 (50.0)	75 (72.8)	0.003	2 (15.4)	12 (46.2)	0.06
**Treatment**						
Remdesivir	209 (8.6)	80 (14.9)	<0.001	11 (2.5)	9 (6.2)	0.03
Steroids	306 (12.5)	118 (22.0)	<0.001	361 (81.7)	119 (81.5)	0.96
IVIG	25 (1.0)	15 (2.8)	0.001	392 (88.7)	128 (87.7)	0.74

IQR = interquartile range; EEG = electroencephalogram; CT = computed tomography; MRI = magnetic resonance imaging; IVIG = intravenous immunoglobulin; [a] = frequency in those who received an EEG; [b] = frequency in those who received a CT; [c] = frequency in those who received a MRI

© 2024 Francoeur C et al. *JAMA Network Open*.
